# Response to Letter to the Editor From Wagner et al: “An Unusually Prolonged Case of FGF23-Mediated Hypophosphatemia Secondary to Ferric Carboxymaltose Use”

**DOI:** 10.1210/jcemcr/luae079

**Published:** 2024-05-14

**Authors:** Ipsa Arora, Alison Kaprove, Ronald Perrone, Lisa Ceglia

**Affiliations:** Division of Endocrinology, Tufts Medical Center, Boston, MA 02111, USA; Division of Nephrology, Tufts Medical Center, Boston, MA 02111, USA; Division of Nephrology, Tufts Medical Center, Boston, MA 02111, USA; Division of Endocrinology, Tufts Medical Center, Boston, MA 02111, USA

We thank Wagner et al for their keen interest in our case report (1, 2). We appreciate their discussion delving into the intricate understanding of the underlying pathophysiology of hypophosphatemia in patients treated with ferric carboxymaltose (FCM) for iron deficiency anemia. In this letter, we address concerns with our case report raised by Wagner et al.

Our patient's prolonged hypophosphatemia subsequent to FCM administration was predominantly driven by “inappropriately normal FGF23” activity. This assessment was based on persistent hypophosphatemia in the setting of a serum intact FGF23 level of 45 pg/mL (reference range <59 pg/mL) drawn 18 months after FCM administration. The “inappropriately normal PTH” level is not the primary driver of the hypophosphatemia but rather a secondary process due to reduced 1α-hydroxylase activity reducing circulating levels of 1,25-dihydroxyvitamin D and lowering calcium absorption. The rationale for treatment with calcitriol was to raise 1,25-dihydroxyvitamin D, thereby stimulating calcium and phosphate absorption in the gut and reabsorption in the kidney tubules. Via negative feedback, increasing 1,25-dihydroxyvitamin D reduced the parathyroid hormone level as we noted in our patient.

We raised the possibility that the FCM dose was a contributing factor to the prolonged hypophosphatemia in our patient because the dose resulted in markedly elevated ferritin levels lasting over 18 months post infusion. According to a study testing FCM infusions in women of similar age to our patient, the rise in ferritin level post FCM correlated with rises in serum FGF23 level and declines in serum phosphate levels, suggesting that greater rises in ferritin increase risk of FGF23-induced hypophosphatemia (3).

The contribution of the PKD1 mutation to the prolonged hypophosphatemia is speculative. Although mice with the mutation demonstrate bone loss (4), there is no evidence that patients with autosomal dominant polycystic kidney disease have low bone mineral density by dual energy x-ray absorptiometry (5, 6). We considered the PKD1 mutation because it may play a role in the regulation of FGF23 expression (7); however, this potential interaction needs additional investigation. The fact that our patient's bone mineral density remained stable over the 3.5 years indicates that treatment with calcitriol successfully prevented significant bone mineral losses.

## Abbreviation

FCM: ferric carboxymaltose
